# Antioxidant Supplementation with Caffeine During Rescue In Vitro Maturation Improves Fertilization and Embryo Development in Women of Advanced Maternal Age

**DOI:** 10.3390/antiox15050555

**Published:** 2026-04-27

**Authors:** Gyungbin Lee, Jin Hee Eum, Tae Hyung Kim, Samuel J. Han, Soyoung Kim, Hee Jun Lee, Youn-Jung Kang

**Affiliations:** 1 Department of Life Science, Graduate School, CHA University, 335 Pangyo-ro, Bundang-gu, Seongnam-si 13488, Republic of Korea; gblee21@chamc.co.kr; 2 CHA University Fertility Center Gangnam, 566 Nonhyeon-ro, Gangnam-gu, Seoul 06135, Republic of Korea; jheum22@chamc.co.kr (J.H.E.); telly@chamc.co.kr (T.H.K.); sysy314@chamc.co.kr (S.K.); alonfo@chamc.co.kr (H.J.L.); 3 Department of Obstetrics and Gynecology, Brian D. Allgood Army Community Hospital, Pyeongtaek-si 17977, Republic of Korea; samuel.han@health.mil; 4 Department of Biochemistry, Research Institute for Basic Medical Science, School of Medicine, CHA University, 335 Pangyo-ro, Bundang-gu, Seongnam-si 13488, Republic of Korea

**Keywords:** rescue in vitro maturation, caffeine, immature oocyte, fertilization rate, embryo development, advanced maternal age

## Abstract

Age-related decline in oocyte quality is closely associated with mitochondrial dysfunction and oxidative imbalance, which disrupt redox-sensitive meiotic signaling and compromise embryo developmental competence. Rescue in vitro maturation (r-IVM) enables the utilization of immature oocytes retrieved during conventional in vitro fertilization (IVF) cycles. However, the developmental potential of r-IVM oocytes remains limited, particularly in women of advanced maternal age. This study evaluated whether transient caffeine supplementation during r-IVM improves the developmental competence of immature human oocytes in clinical assisted reproduction technology cycles. Immature oocytes obtained during conventional IVF were cultured with or without short-term caffeine exposure during r-IVM prior to standard culture conditions. After maturation, metaphase II oocytes underwent intracytoplasmic sperm injection, and embryonic development was assessed by fertilization rate, day 3 good-quality embryo formation, and blastocyst development. Although caffeine supplementation did not significantly affect nuclear maturation rates, it significantly increased fertilization efficiency and the proportion of good-quality embryos compared with controls. These effects were most pronounced in women aged ≥37 years. Time-lapse morphokinetic analysis further revealed more synchronized developmental kinetics in embryos derived from caffeine-treated oocytes, resembling those derived from in vivo-matured oocytes. Collectively, these findings suggest that transient caffeine exposure during r-IVM enhances post-fertilization developmental competence. The underlying mechanisms remain to be elucidated, and future studies are required to determine whether redox-sensitive meiotic pathways and mitochondrial function are involved.

## 1. Introduction

The global trend toward delayed childbearing has led to a substantial increase in infertility associated with advanced maternal age (AMA) and diminished ovarian reserve (DOR) [1]. Ovarian aging is characterized not only by depletion of the follicular pool but also progressive deterioration in oocyte quality [2]. Accumulating evidence indicates that age-related decline in oocyte competence is strongly associated with mitochondrial dysfunction, impaired bioenergetic capacity, altered redox homeostasis, and dysregulated meiotic signaling [3,4]. These molecular alterations compromise fertilization efficiency and embryonic developmental potential, thereby limiting the success of assisted reproductive technology (ART).

During conventional in vitro fertilization (IVF), controlled ovarian hyperstimulation enables retrieval of multiple oocytes; however, 15–30% remain immature at the germinal vesicle (GV) or metaphase I (MI) stage at the time of oocyte retrieval [5]. This proportion is significantly higher in AMA patients and poor ovarian responders [6]. Such immature oocytes are commonly discarded, resulting in a further reduction in the number of embryos available for transfer. Rescue in vitro maturation (r-IVM), in which immature oocytes obtained during conventional IVF cycles are cultured to reach metaphase II (MII) in vitro, represents a clinically relevant strategy to expand the usable oocyte cohort, particularly in patients with limited ovarian reserve [7]. Nevertheless, developmental outcomes of r-IVM oocytes remain inferior to those matured in vivo. This limitation is largely attributed to incomplete cytoplasmic maturation and inadequate coordination between meiotic progression and mitochondrial redox regulation [8].

Oocyte maturation requires tightly synchronized activation of maturation-promoting factor (MPF) and mitogen-activated protein kinase (MAPK) signaling pathways [9]. These pathways are redox-sensitive and closely linked to mitochondrial function. Mitochondria supply ATP required for spindle assembly and chromosomal segregation but also generate ROS as byproducts of oxidative phosphorylation [3,10]. While physiological ROS levels act as signaling mediators, excessive ROS production disrupts mitochondrial membrane potential, induces lipid and DNA damage, and impairs embryonic development [11]. In vitro culture conditions may exacerbate oxidative imbalance, particularly in oocytes from AMA or DOR patients, whose antioxidant capacity is often diminished.

Caffeine (1,3,7-trimethylxanthine) is a bioactive methylxanthine compound traditionally recognized as a non-selective phosphodiesterase inhibitor and adenosine receptor antagonist [12,13]. Beyond its role in cyclic AMP signaling and cell cycle regulation, caffeine has been reported to exert antioxidant properties through modulation of ROS production, mitochondrial function, and redox-sensitive signaling pathways [14,15]. Experimental studies in mammalian oocytes have demonstrated that caffeine can transiently regulate MPF and MAPK activity, preserve spindle integrity, and improve mitochondrial distribution, and enhance blastocyst formation rates [16,17]. These effects suggest that caffeine may function not only as a meiotic regulator but also as a redox-modulating agent capable of supporting cytoplasmic maturation. However, the antioxidant-associated effects of caffeine in human r-IVM, particularly in oocytes from AMA or DOR patients, remain insufficiently characterized.

Given the central involvement of mitochondrial dysfunction and oxidative stress in age-related oocyte decline, we hypothesized that transient caffeine supplementation during r-IVM would enhance oocyte developmental competence by modulating redox-sensitive meiotic signaling and improving mitochondrial function. To address this, we evaluated the effects of short-term caffeine exposure on maturation, fertilization, and blastocyst formation rates in immature oocytes retrieved during conventional IVF cycles in patients with AMA or DOR. This study aims to determine whether pharmacological modulation using caffeine can improve r-IVM outcomes and expand the therapeutic potential of ART in patients with limited ovarian reserve.

## 2. Materials and Methods

### 2.1. Study Design and Patient Population

This retrospective cohort study was conducted at CHA University Fertility Center Gangnam and approved by the Institutional Review Board (IRB No. 2024-06-012, approval date: 14 August 2024). The requirement for informed consent was waived due to the retrospective nature of the study. Patients who underwent conventional IVF cycles between June 2023 and May 2024 were screened. Among 837 infertile patients in whom immature oocytes were identified after oocyte denudation, r-IVM was performed. Of these, 449 patients had oocytes that reached the MII stage following r-IVM and subsequently underwent intracytoplasmic sperm injection (ICSI). Exclusion criteria were (1) absence of GV oocyte, (2) use of cryopreserved oocytes, and (3) cycles involving surgically retrieved sperm, including testicular sperm extraction (TESE) or percutaneous epididymal sperm aspiration (PESA). To improve group comparability and reduce potential selection bias, additional eligible patients were included in the control group based on the same inclusion and exclusion criteria. Due to the retrospective design and potential limitations in statistical power within subgroups (e.g., PCOS, endometriosis, and male factor), formal multivariable or stratified analyses were not performed.

### 2.2. Controlled Ovarian Hyperstimulation, Oocyte Retrieval, and r-IVM Protocol

All patients underwent controlled ovarian hyperstimulation (COH) using either a gonadotropin-releasing hormone (GnRH) agonist protocol (Lorelin Depot, Dong Kook, Seoul, Republic of Korea) or an antagonist (Cetrotide^®^, Merck, Darmstadt, Germany or Orgalutran^®^, MSD, Kenilworth, NJ, USA or Ganilever™, LG Chem, Seoul, Republic of Korea) for pituitary suppression. Final oocyte maturation was triggered with recombinant human chorionic gonadotropin (hCG) (Ovidrel^®^, Merck, Darmstadt, Germany), and cumulus-oocyte complexes (COCs) were retrieved 36–38 h later. The retrieved oocytes were cultured in a fertilization medium (Quinn’s Advantage™ Protein Plus, CooperSurgical, Trumbull, CT, USA), and 2 h after OPU the COCs were denuded using enzymes (Hyaluronidase, CooperSurgical, Trumbull, CT, USA) and repeated pipetting. The nuclear maturation status of all denuded oocytes was assessed and classified as GV, MI, or MII stage. In the control group, GV-stage oocytes were cultured in in-house IVM media with 10 uL of hCG, melatonin, E_2_ and FSH added to 10 mL of culture medium (G-2™ PLUS, Vitrolife, Gothenburg, Sweden) respectively, and for the caffeine group, it was cultured by adding caffeine to in-house IVM media. Oocytes at the MI stage were not subjected to r-IVM; these oocytes were maintained under standard culture conditions and allowed to mature spontaneously to the MII stage after approximately 24 h (designated as the MI-MII groups). Patients were classified into the caffeine treatment group (C8960; Sigma-Aldrich Chemical Company, Burlington, MA, USA) or the control group (IVM without caffeine) according to the treatment they received. In the caffeine treatment group, oocytes were cultured in an in-house IVM medium supplemented with 1.25 mM caffeine for 4 h, then transferred to standard IVM conditions. Oocyte maturity was assessed the following day, and ICSI was performed on MII oocytes. As this study involved human oocytes, the concentration range associated with enhanced blastocyst formation (1 to 2.5 mM) reported in previous studies was used as a reference [18,19,20]. Specifically, 1.25 mM caffeine was selected based on prior evidence demonstrating improved blastocyst formation and safe application in human somatic cell nuclear transfer models [21]. Given the practical limitations in testing multiple concentrations in clinical oocytes, this concentration was adopted as a representative, outcome-based condition. In addition, the average time to germinal vesicle breakdown (GVBD) in human oocytes has been reported to be approximately 3.7 h; therefore, a 4-h incubation period was applied to facilitate synchronization of cytoplasmic maturation and assess its impact on subsequent embryonic development [22].

### 2.3. Embryo Culture and Evaluation Following ICSI

**Figure 1:**
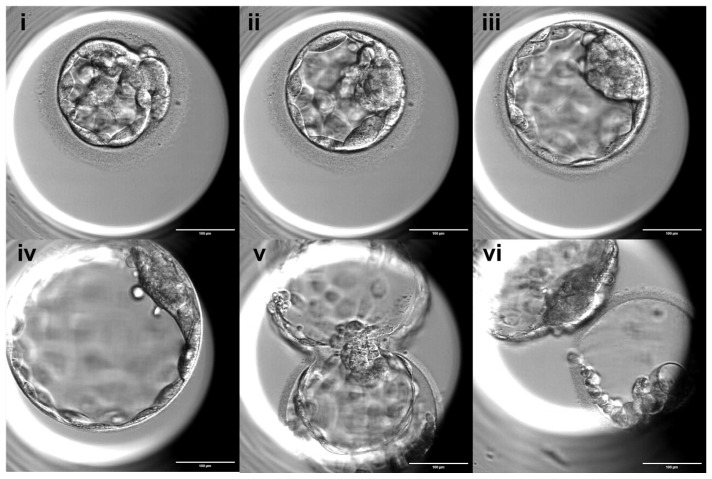
Classification of blastocyst expansion stages. Blastocyst development was categorized into six stages: (**i**) early blastocyst, in which the blastocoel cavity occupies less than half of the embryo volume; (**ii**) mid blastocyst, in which the blastocoel cavity occupies more than half of the embryo volume; (**iii**) full blastocyst, in which the blastocoel cavity completely fills the embryo; (**iv**) expanded blastocyst, characterized by an enlarged blastocoel cavity and thinning of the zona pellucida; (**v**) hatching blastocyst, in which trophectoderm cells begin to herniate through the zona pellucida; and (**vi**) hatched blastocyst, in which the embryo has completely escaped from the zona pellucida. Scale bar: 100 µm.

**Figure 2:**
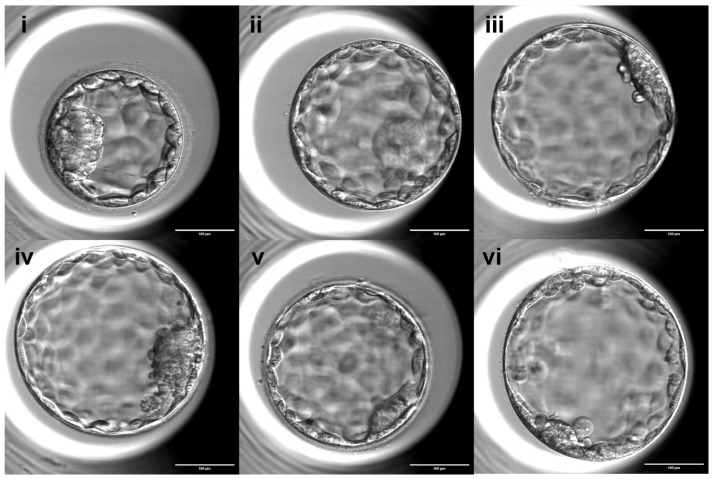
The quality of the inner cell mass (ICM) and trophectoderm (TE) cells. The ICM was assessed as follows: (**i**) A, many compacted cells, tightly packed; (**ii**) B, several loosely grouped cells; or (**iii**) C, very few cells. The TE was assessed as follows: (**iv**) A, many cells forming a cohesive epithelium; (**v**) B, few cells forming a loose epithelium; or (**vi**) C, very few large cells. Scale bar: 100 µm.

For ICSI, sperm cells were placed in 7% Polyvinylpyrrolidone (PVP, CooperSurgical, USA) containing medium and selected based on morphology and motility under a conventional light microscope (Ti2, Nikon, Nishioi, Japan). Selected spermatozoa were then injected into the cytoplasm of oocytes. All injected oocytes were individually cultured in cleavage medium with a total of 20 uL double droplet (Quinn’s Advantage™ Protein Plus, CooperSurgical, Trumbull, CT, USA) and covered with paraffin oil (OVOIL™, Vitrolife, Gothenburg, Sweden). Oocytes from a subset of patients were cultured in a tri-gas incubator (K-Systems™, CooperSurgical, Trumbull, CT, USA or Heracell™, Thermo Fisher Scientific, Waltham, MA, USA) at 5% O_2_ and 6% CO_2_, while others were cultured in a time-lapse incubator (EmbryoScope™, Vitrolife, Gothenburg, Sweden). Normal fertilization was defined by the presence of two pronuclei (2PN) 16–18 h after ICSI, whereas the presence of a single pronucleus or three or more pronuclei was classified as abnormal fertilization. On day 3, 48 h after pronuclear detection, the cleavage development rate was evaluated, and embryos were transferred to blastocyst medium (Quinn’s Advantage™ Protein Plus, CooperSurgical, Trumbull, CT, USA). Good-quality cleavage stage embryos were defined as those with 6 or more blastomeres and less than 20% fragmentation. Blastocyst morphology, including expansion stage, inner cell mass (ICM), and trophectoderm (TE) quality, was assessed by experienced embryologists according to standardized criteria routinely used in our center based on the Gardner grading system, which assesses the degree of expansion (graded 1 to 6), as well as the quality of the ICM and TE cells (graded A to C). Blastocyst expansion was classified into 6 stages: 1, Early blastocyst: the blastocoel cavity occupies less than half of the embryo’s volume; 2, Mid blastocyst: the blastocoel cavity occupies more than half of the embryo’s volume; 3, Full blastocyst: the blastocoel cavity completely fills the embryo; 4, Expanded blastocyst: the blastocoel cavity is larger than in the early stages and the zona pellucida begins to thin; 5, Hatching blastocyst: the trophectoderm cells begin to herniate through the zona pellucida, and 6, Hatched blastocyst: the blastocyst has completely escaped from the zona pellucida (Figure 1). The ICM was assessed as follows: A, many compacted cells, tightly packed; B, several loosely grouped cells; or C, very few cells. The TE was assessed as follows: A, many cells forming a cohesive epithelium; B, few cells forming a loose epithelium; or C, very few large cells (Figure 2). As this was a retrospective study, the assessments were not performed in a blinded manner.

### 2.4. Time-Lapse Culture and Morphokinetics

The time-lapse incubator (EmbryoScope™, Vitrolife, Gothenburg, Sweden) is equipped with an embryo monitoring system that captures images every 10 min at seven different focal planes. Embryo scoring was determined by the judgment of two experienced embryologists. The following morphokinetic parameters were recorded: time of pronuclei fading (tPNf), defined as the last frame in which both pronuclei are no longer visible; cleavage times from the 2-cell to the 9-cell stage (from t2 to t9, respectively), defined as the first frame at which the corresponding number of distinct blastomeres is observed; first evidence of compaction (tSC), defined as the first frame in which a discernible blastocoel cavity appears; initiation of blastulation (tSB), defined as the first frame showing the appearance of a discernible blastocoel cavity; full blastocyst stage, last frame before zona thinning starts (tB), defined as the last frame before the onset of zona pellucida thinning with a fully formed blastocoel cavity; initiation of expansion, first frame of zona thinning (tEB), defined as the first frame showing expansion of the blastocoel cavity accompanied by visible thinning of the zona pellucida [23].

### 2.5. Statistical Analysis

The study employed a paired analysis design. Results are presented as the mean ± standard deviation (SD) or counts with percentages [n (%)]. Comparisons between groups, including patient’s age, anti-Mullerian hormone (AMH) levels, antral follicle count (AFC), body mass index (BMI) were performed using Student’s *t*-test. Chi-square tests were used to assess infertility indications, differences in fertilization rates and the proportion of good-quality embryos between groups in oocyte level. Mann–Whitney U tests were used to assess differences in fertilization rates and the proportion of good-quality embryos between groups at patient level. Statistical analysis was conducted using IBM Statistical Packages for the Social Sciences Version 29.0 (SPSS, IBM Corp., Armonk, NY, USA), Graphpad 11 (GraphPad Software, Boston, MA, USA), with a *p*-value of less than 0.05, considered statistically significant.

## 3. Results

### 3.1. Characterization of the Study Population

**Figure 3:**
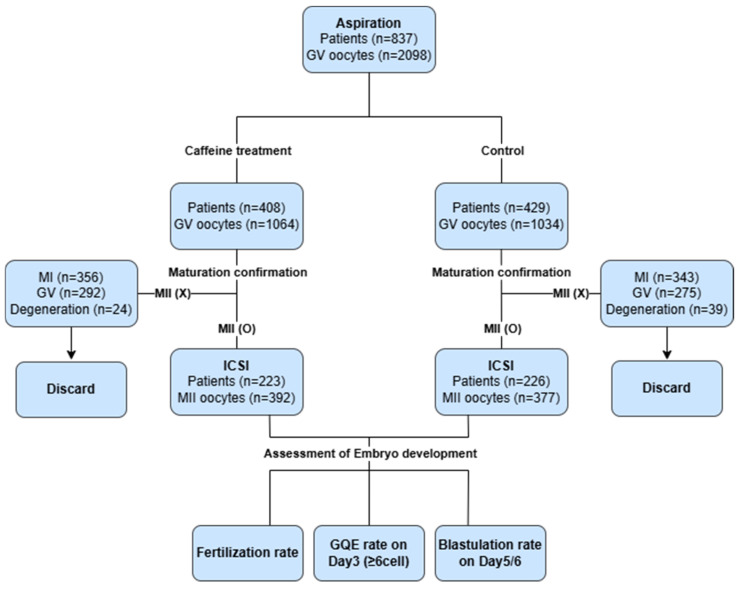
Procedures of caffeine treatments. MII, metaphase II; MI, metaphase I; GV, germinal vesicle; Degeneration, characterized by lysis of the oocyte. A total of 837 patients, 2098 GV oocytes identified during conventional IVF cycles were included. Of these, 408 patients were included in the caffeine-treated group and 429 to the control group. Following r-IVM, 449 patients achieved at least one MII-stage oocyte and subsequently underwent ICSI. Embryonic development was assessed by fertilization rate, day 3 good-quality embryo formation, and blastocyst development.

A total of 837 patients with immature oocytes identified during conventional IVF cycles were included. Of these, 408 patients were included in the caffeine-treated group and 429 to the control group. Following r-IVM, 449 patients achieved at least one MII-stage oocyte and subsequently underwent ICSI (caffeine-treated, n = 223; control, n = 226) (Figure 3). Baseline characteristics, including maternal age, serum AMH levels, AFC, BMI and infertility etiology were not significantly different between groups (Table 1). Although no statistically significant difference in infertility etiology was observed between groups (*p* = 0.082), this does not necessarily indicate true comparability.

### 3.2. Effect of Caffeine Supplementation on Oocyte Nuclear Maturation

**Figure 4:**
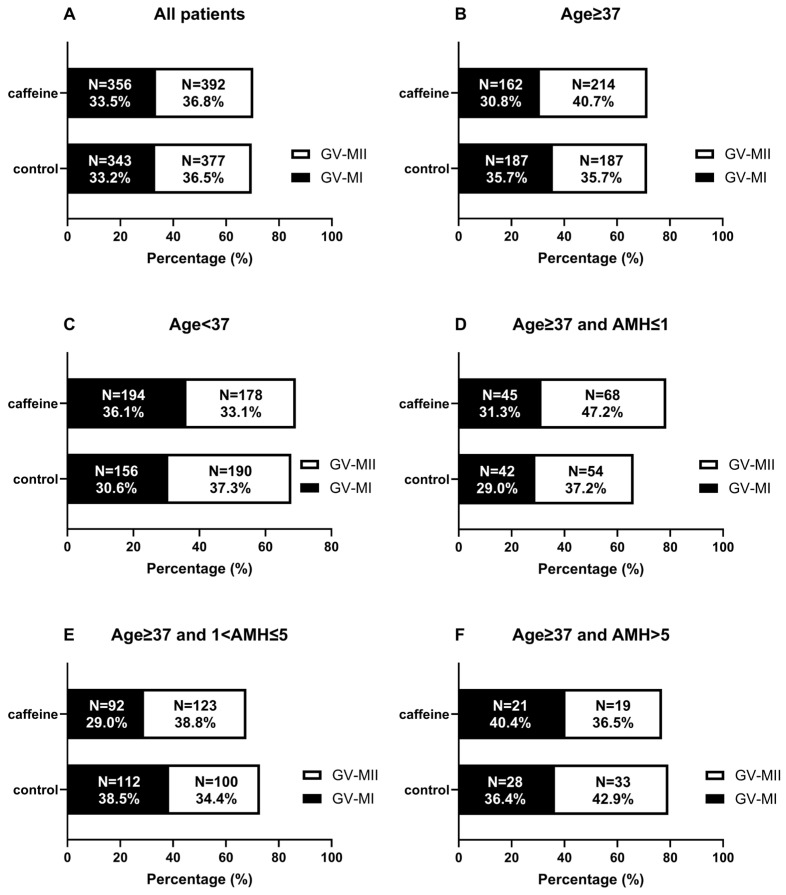
Effects of caffeine treatment on oocyte maturation. Comparison of the rates of maturation from GV stage oocytes (GV–MI and GV–MII maturation) between the control and caffeine-treated groups across the entire cohort (**A**), as well as in subgroups based on maternal age (**B**,**C**) and AMH levels (**D**–**F**).

In the overall cohort, the proportion of GV-stage oocytes progressing to the MII stage following r-IVM was comparable between the control and caffeine-treated group (36.5%, 377/1034 vs. 36.8%, 392/1064, respectively). The proportion of oocytes arrested at the MI stage was likewise similar (33.2%, 343/1034 vs. 33.5%, 356/1064) (Figure 4A). Age-stratified analysis revealed that in patients aged ≥37 years, the MII maturation rate was numerically higher in the caffeine-treated group compared with control group (35.7%, 187/524 vs. 40.7%, 214/526), (Figure 4B). Among patients aged <37 years, the control group demonstrated a slightly higher maturation rate than the caffeine-treated group (37.3%, 190/510 vs. 33.1%, 178/538) (Figure 4C). When analyzed according to ovarian reserve in the AMA group (control group (n = 2) and caffeine group (n = 4) without AMH level are excluded from subgroup analysis according to AMH), patients with AMH ≤ 1 ng/mL and 1 < AMH ≤ 5 exhibited a higher MII maturation rate in the caffeine-treated group compared with the control group (37.2%, 54/145 vs. 47.2%, 68/144, 34.4%, 100/291 vs. 38.8%, 123/317, respectively) (Figure 4D,E). AMH > 5 ng/mL exhibited a higher MII maturation rate in the control group compared with the caffeine-treated group (42.9%, 33/77 vs. 36.5%, 19/52) (Figure 4F). Across both the overall cohort and stratified analyses, caffeine supplementation was not associated with significant changes in nuclear maturation rates of GV-stage oocytes.

### 3.3. Caffeine Supplementation Improves Fertilization and Early Embryonic Development in the Overall Cohort

**Figure 5:**
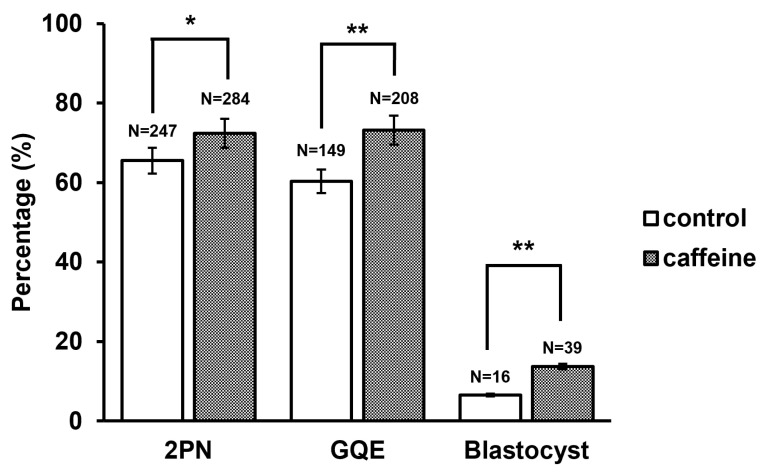
Effect of caffeine treatment on embryo developmental progression. The percentage of embryos at 2PN, day 3 GQE, and blastocyst stage in control and caffeine-treated groups. The numbers above the bars indicate the total number of embryos analyzed in each condition. Data are presented as percentages of embryos at each developmental stage. *P* values were calculated using the Chi-square tests. Statistical significance is indicated as * *p* < 0.05 and ** *p* < 0.01 compared with the control group.

Although nuclear maturation rates were comparable between groups, caffeine supplementation was associated with improved ICSI developmental outcomes. The fertilization rate, defied by the presence of 2PN, was significantly higher in the caffeine-treated group compared with the control group (72.4% vs. 65.5%, *p* = 0.038). In addition, the proportion of good-quality embryos (GQEs) on day 3, defined as 6 or more blastomeres and less than 20% fragmentation, was significantly increased in the caffeine-treated group (73.2% vs. 60.3%, *p* = 0.002). The blastocyst formation rate on day 5/6 was significantly higher in the caffeine-treated group than in controls (13.7% vs. 6.5%, *p* = 0.006) (Figure 5). In addition, to address the potential clustering effect we conducted non-parametric statistics to confirm the characteristics of each patient group. There was no significant difference in fertilization outcomes between the caffeine-treated and control groups (median 100.0 vs. 100.0, *p* = 0.345). However, significant differences were observed in GQE (median 100.0 vs. 100.0, *p* = 0.011) and blastocyst formation (median 0.0 vs. 0.0, *p* = 0.006) (Table 2). A similar trend was observed in the per-patient analysis, although the statistical significance was attenuated in fertilization rate. Collectively, these data suggest that transient caffeine supplementation during r-IVM was associated with improved fertilization efficiency and early embryonic developmental potential, despite no significant differences in nuclear maturation rates.

### 3.4. Age-Stratified Analysis of Embryonic Development

**Figure 6:**
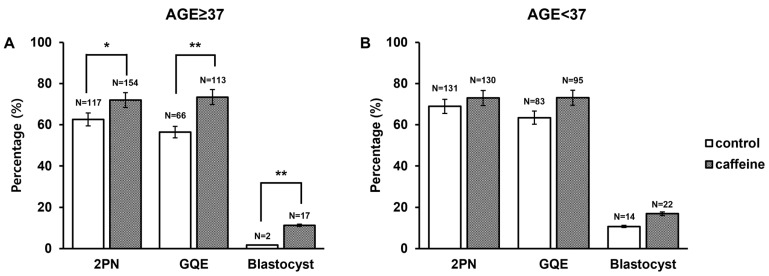
Effect of caffeine treatment on embryo developmental progression according to maternal age. The percentage of embryos at 2PN, day 3 GQE, and blastocyst stage in control and caffeine-treated groups in women aged ≥37 years (**A**) and <37 years (**B**). The numbers above the bars indicate the total number of embryos analyzed in each condition. Data are presented as percentages of embryos at each developmental stage. Statistical significance is indicated as * *p* < 0.05 and ** *p* < 0.01 compared with the control group.

In patients aged ≥37 years, caffeine supplementation was associated with significantly improved fertilization (72.0% vs. 62.6%, *p* = 0.045) and day 3 GQE rates (73.4% vs. 56.4%, *p* = 0.003) compared with controls. A significantly higher blastocyst formation rate was observed in the caffeine-treated group (11.3% vs. 1.7%, *p* = 0.003) (Figure 6A). In contrast, among aged <37 years, no statistically significant differences were observed between groups for fertilization (73.0% vs. 68.9%, *p* = 0.388), day 3 embryo quality (73.1% vs. 63.4%, *p* = 0.092), or blastocyst formation (16.9% vs. 10.7%, *p* = 0.144) (Figure 6B). Therefore, the beneficial effects of caffeine supplementation were primarily observed in the AMA subgroup.

### 3.5. AMH-Stratified Analysis in Patients Aged ≥37 Years

**Figure 7:**
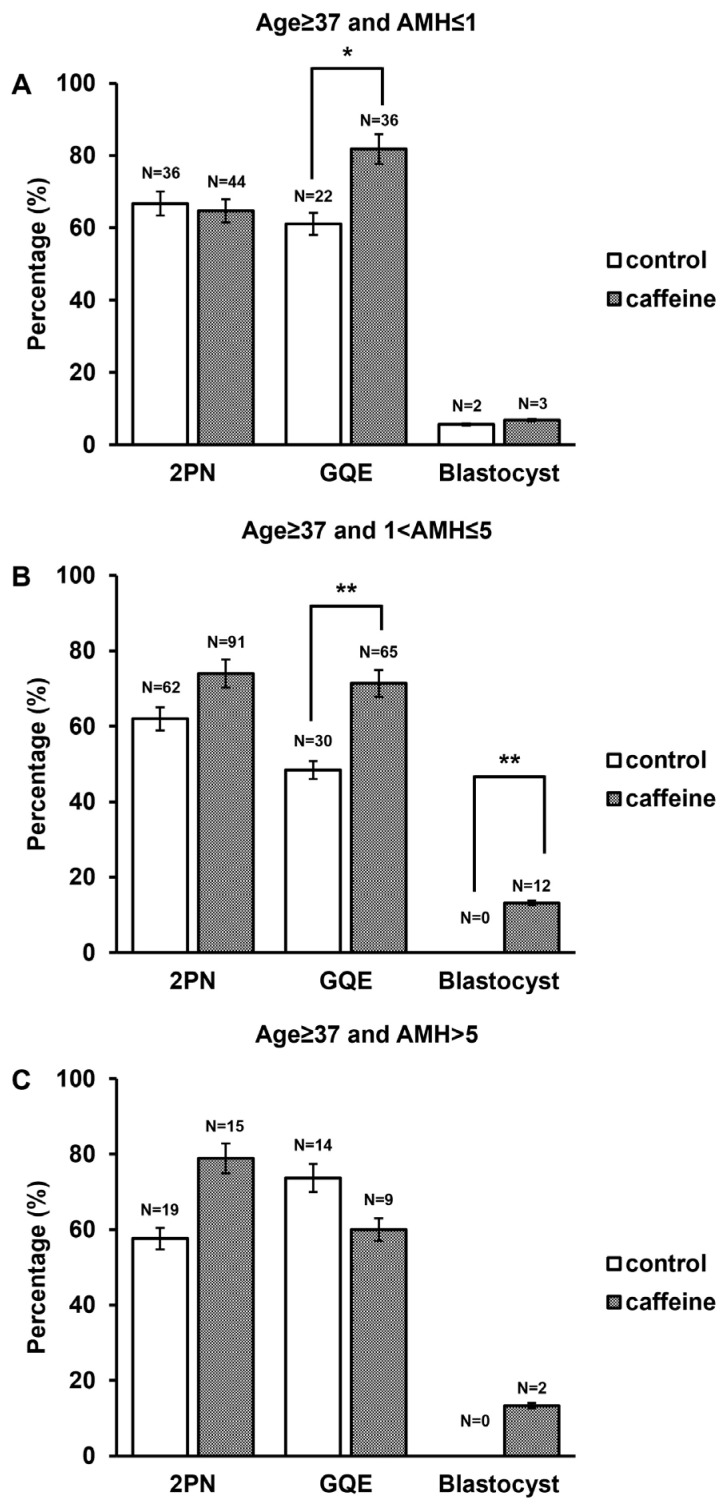
Effect of caffeine supplementation on embryo developmental outcomes in women aged ≥37 years according to AMH levels. The percentage of embryos at 2PN, day 3 GQE, and blastocyst stage in control and caffeine-treated groups in women aged ≥37 years stratified by AMH ≤ 1 ng/mL (**A**), 1–5 ng/mL (**B**), >5 ng/mL (**C**). The numbers above the bars indicate the total number of embryos analyzed in each condition. Data are presented as percentages of embryos at each developmental stage. Statistical significance is indicated as * *p* < 0.05 and ** *p* < 0.01 compared with the control group.

To further delineate the interaction between ovarian reserve and caffeine supplementation independent of maternal age, a stratified analysis was performed exclusively in patients aged ≥37 years, with additional subdivision according to serum AMH levels (≤1 ng/mL, 1–5 ng/mL, and >5 ng/mL). Among patients with low ovarian reserve (AMH ≤ 1 ng/mL), fertilization rates were comparable between groups (64.7% vs. 66.7%, *p* = 0.821). The day 3 GQE rate was significantly higher in the caffeine-treated group (81.8% vs. 61.1%, *p* = 0.039), whereas blastocyst formation did not differ significantly (6.8% vs. 5.6%, *p* = 0.816) (Figure 7A). In patients with intermediate ovarian reserved (AMH 1–5 ng/mL), caffeine supplementation was associated with significantly improved ICSI outcomes. Fertilization rates were numerically higher in the caffeine-treated group compared with controls (74.0% vs. 62.0%, *p* = 0.055). Moreover, the day 3 GQE rate was significantly increased in the caffeine-treated group (71.4% vs. 48.4%, *p* = 0.004). Blastocyst formation occurred more frequently in the caffeine-treated group (13.2% vs. 0.0%, *p* = 0.003) (Figure 7B). Furthermore, among patients with relatively higher AMH levels (>5 ng/mL), fertilization (78.9% vs. 57.6%, *p* = 0.119) and blastocyst formation (13.3% vs. 0%, *p* = 0.101) were numerically higher in the caffeine-treated group, although these differences were not statistically significant (Figure 7C). These findings indicate that, within the AMA population, the beneficial effect of caffeine supplementation was most pronounced in patients with intermediate ovarian reserve, suggesting a potential interaction between ovarian reserve status and caffeine-mediated enhancement of developmental competence.

### 3.6. Time-Lapse Morphokinetic Analysis

**Figure 8:**
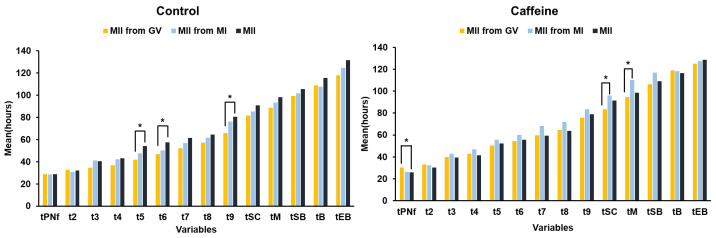
Embryo developmental kinetics according to oocyte maturation stage in control and caffeine-treated groups. The mean time (hours) to reach each developmental stage in embryos derived from oocytes at different maturation stages in control (**left**) and caffeine-treated (**right**) groups. MII obtained from GV oocytes (yellow), MII obtained from MI oocytes (blue), and in vivo-matured MII oocytes (black). Developmental parameters include time to PN fading (tPNf), cleavage stages (t2–t9), time to start of compaction (tSC), morula (tM), start of blastulation (tSB), blastocyst formation (tB), and expanded blastocyst (tEB). Detailed definitions of these parameters are described in Section 2.4. Data are presented as mean time of embryos at each developmental stage. Statistical significance between groups in indicated by * *p* < 0.05.

**Figure 9:**
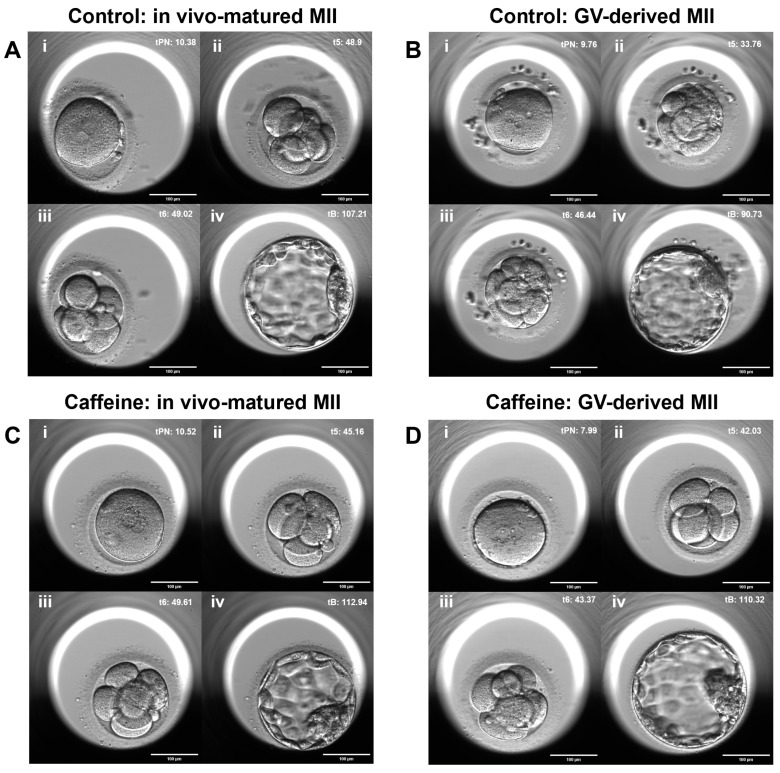
Representative time-lapse images of embryonic development derived from in vivo-matured and r-IVM oocytes under control and caffeine-treated conditions. Representative morphokinetic images obtained using an EmbryoScope time-lapse imaging system illustrating early embryonic development from different oocyte maturation conditions. The upper panels represent the control group (**A**,**B**) and the lower panels represent the caffeine-treated group during r-IVM (**C**,**D**). Sequential developmental stages are indicated as: (i) pronuclear state (tPN), (ii) 5-cell stage (t5), (iii) 6-cell stage (t6), and (iv) blastocyst formation (tB). Scale bar: 100 µm.

Time-lapse analysis revealed distinct developmental kinetic patterns between groups (Table 3 and Figure 8). In the control group, GV-derived MII oocytes exhibited accelerated early cleavage timing compared with in vivo-matured MII oocytes, including significantly shorter time to the 5-cell stage (t5: 41.9 ± 8.6 h vs. 54.2 ± 12.1 h; *p* = 0.011) and 6-cell stage (t6: 46.8 ± 8.7 h vs. 57.4 ± 11.9 h; *p* = 0.023). In contrast, embryos derived from caffeine-treated oocytes demonstrated more synchronized and consistent morphokinetic profiles, with timing patterns comparable to those of in vivo-matured MII oocytes. Notably, the differences among GV-derived, MI-derived, and in vivo-matured MII oocytes were reduced under caffeine treatment, indicating improved synchronization of developmental kinetics across groups. Early cleavage was less accelerated, and compaction and blastulation progression appeared more coordinated. Representative time-lapse images obtained using the EmbryoScope system further illustrate these differences (Figure 9). In the control group, embryos derived from GV-mature MII oocytes exhibited accelerated cleavage dynamics relative to embryo derived from in vivo-matured MII oocytes. In a representative control patient, embryos derived from GV-derived MII oocytes displayed accelerated cleavage compared with embryos derived from in vivo-matured MII oocytes, reaching the 5-cell stage earlier (t5: 48.9 h vs. 33.76 h) (Figure 9(Ai,Aii,Bi,Bii)) and the 6-cell stage earlier (t6: 49.02 h vs. 46.44 h) (Figure 9(Aiii,Biii)). In contrast, in embryos in the caffeine-treated group, more similar developmental timing between in vivo-matured and GV-derived MII oocytes were observed, indicating a more coordinated progression of early cleavage events (Figure 9C,D). Collectively, these observations indicate that caffeine supplementation during r-IVM modifies early embryonic developmental kinetics, promoting morphokinetic patterns that more closely resemble those of embryos derived from in vivo-matured oocytes. This suggests improved coordination between nuclear and cytoplasmic maturation processes following caffeine exposure.

### 3.7. Translational Clinical Outcomes of Caffeine-Supplemented r-IVM

**Figure 10:**
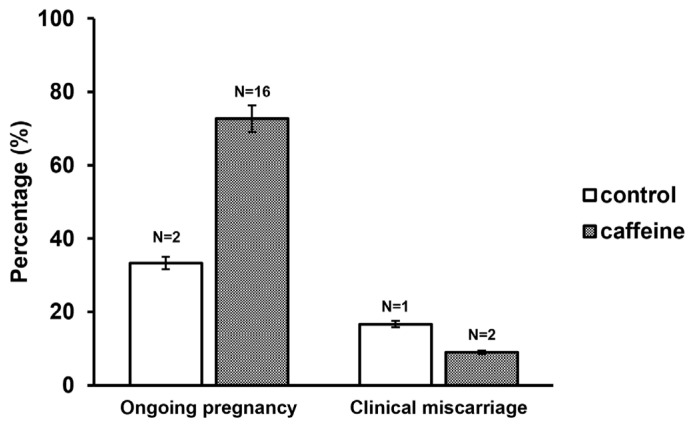
Clinical outcomes following embryo transfer in control and caffeine-treated groups. The percentage of ongoing pregnancy and clinical miscarriage in the control and caffeine-treated groups. The numbers above the bars indicate the total number of patients analyzed in each condition. Data are presented as percentages of clinical outcomes.

To evaluate whether the embryological improvement observed with caffeine supplementation translated into clinical benefit, pregnancy outcomes were analyzed in patients who underwent embryo transfer using GV-derived embryos. Among transferred cycles, the ongoing pregnancy rate was higher in the caffeine-treated group compared with controls (72.7% vs. 33.3%, *p* = 0.074). Although this difference did not reach statistical significance, a clear numerical increase was observed. Clinical miscarriage rates were lower in the caffeine-treated group compared with controls, resulting in the proportion of cycles without pregnancy was reduced (18.2% vs. 50.0%). Given the limited sample size in the transfer cohort, statistical power was insufficient to detect definitive significance; however, the directionality of the outcomes suggests a potential clinical advantage associated with caffeine supplementation during r-IVM (Figure 10).

## 4. Discussion

Oocyte development competence depends on precise coordination between nuclear progression and cytoplasmic maturation, processes that are tightly linked to mitochondrial function and intracellular redox balance [4,18]. Age-related decline in oocyte quality is characterized by impaired mitochondrial bioenergetics, increased oxidative stress, and dysregulated meiotic signaling, all of which contribute to reduced fertilization capacity and compromised embryo development. r-IVM further challenges this balance, as immature oocytes are exposed to an artificial culture environment that may exacerbate redox instability, particularly in women with AMA or DOR.

In this study, transient caffeine supplementation during r-IVM did not significantly alter nuclear maturation rates but was associated with improved fertilization efficiency and early embryonic development. Across the overall cohort, caffeine-treated oocytes exhibited high 2PN formation rates, along with increased day 3 GQE and blastocyst formation rates. The presence of extreme values (e.g., 0% or 100%) at the patient level likely contributed to the observed statistical differences despite similar median values. Age-stratified analysis demonstrated that these effects were predominantly observed in patients aged ≥37 years, in whom fertilization, cleavage-stage quality, and blastocyst formation were significantly enhanced. Further stratification within the AMA cohort revealed that the magnitude of benefit was greatest in patients with intermediate AMH (1–5 ng/mL), suggesting that caffeine responsiveness may depend on residual ovarian reserve. The absence of differences in MII rates indicates that caffeine does not primarily enhance nuclear maturation. Rather, the improved post-fertilization outcomes suggest enhanced cytoplasmic competence. Cytoplasmic maturation encompasses mitochondrial redistribution, ATP accumulation, mRNA storage, and spindle assembly readiness, which processes essential for accurate chromosomal segregation and early embryonic cleavage [24,25]. Asynchronous meiotic resumption without adequate cytoplasmic preparation has been associated with spindle defects and aberrant cleavage dynamics [26]. Thus, improved fertilization and embryo quality in the absence of increased MII rates support the hypothesis that caffeine facilitates coordinated maturation rather than accelerating meiotic progression.

Time-lapse morphokinetic analysis further supports these findings. In the control group, GV-derived embryos exhibited accelerated early cleavage kinetics relative to in vivo-matured MII oocytes, suggesting premature cell cycle progression. In contrast, embryos derived from caffeine-treated oocytes displayed developmental timing patterns more closely resembling those of in vivo-matured oocytes, with improved synchronization of cleavage, compaction, and blastulation. Excessively rapid early cleavage has been linked to impaired cytoplasmic–nuclear coordination; therefore, normalization of developmental kinetics may reflect improved cytoplasmic readiness. Mechanistically, caffeine functions as a non-selective phosphodiesterase inhibitor and adenosine receptor antagonist, modulating cyclic-AMP levels and meiotic checkpoint signaling [12,13]. MPF and MAPK pathways, which govern meiotic progression, are sensitive to intracellular redox conditions and mitochondrial activity. Beyond cell cycle modulation, caffeine exhibits antioxidant-associated properties, including attenuation of ROS accumulation and stabilization of mitochondrial function in various cellular systems [14]. Given that mitochondria are both ATP generators and primary sources of ROS, particularly in aging oocytes, transient caffeine exposure during r-IVM may help maintain redox-sensitive signaling within a physiologic range, thereby supporting cytoplasmic maturation and developmental competence.

The preferential benefit observed in AMA patients is consistent with the concept that aged oocytes exhibit increased oxidative vulnerability. Intermediate-reserve patients (AMH 1–5 ng/mL) may retain sufficient mitochondrial capacity to respond to redox modulation, whereas severely compromised or highly preserved ovarian reserve states may exhibit differential responsiveness. Although the precise molecular mechanisms remain incompletely defined, the present data suggest that caffeine may enhance cytoplasmic regulatory pathways involved in accurate cell division during early embryogenesis in AMA patients.

From a clinical perspective, these findings may have important implications. Errors in chromosomal segregation during early embryogenesis are a major contributor to aneuploidy, including trisomy, particularly in older patients [17]. Interventions that improve cytoplasmic maturation and spindle regulation could help reduce these risks and improve overall reproductive outcomes. Moreover, the relative simplicity and low cost of caffeine supplementation make it a potentially accessible adjunct to existing ART protocols for clinics treating patients with AMA or diminished ovarian reserve.

This study has several limitations. First, given the retrospective design and the inclusion of clinically heterogeneous infertility etiologies, residual confounding cannot be entirely excluded despite multivariable adjustments and subgroup analyses. Future prospective studies with more homogeneous or stratified populations are warranted to validate our findings. Second, direct measurements of oxidative stress markers, mitochondrial membrane potential, and ATP production were not performed. Therefore, the proposed redox-associated mechanism remains speculative. Further prospective studies incorporating detailed assessments of mitochondrial function and intracellular oxidative status are required to confirm the mechanistic basis of our observations. Finally, although morphological assessments were conducted using standardized criteria, the lack of blinding may introduce potential bias in these subjective evaluations. Consequently, the proposed redox-associated mechanism remains speculative. Prospective studies incorporating qualitative assessment of mitochondrial function and intracellular oxidative status are required to validate the mechanistic basis of these findings.

## 5. Conclusions

In summary, transient caffeine supplementation during r-IVM was associated with improved fertilization efficiency, enhanced early embryonic development, and more physiologically synchronized morphokinetics, particularly in women of AMA. These findings support the concept that modulation of redox-sensitive meiotic signaling may represent a rational strategy to optimize r-IVM outcomes and improve ART efficacy in patients with age-related oocyte compromise.

## Figures and Tables

**Table 1:** Patient characteristics.

Characteristic	Caffeine-Treated Group (n = 408)	Control Group (n = 429)	*p*-Value
Maternal Age (years)	37.9 ± 4.2	37.8 ± 4.3	0.351
BMI (kg/m^2^)	21.7 ± 3.2	21.9 ± 3.2	0.467
Number of IVF cycles	3.6 ± 4.0	3.8 ± 3.4	
AFC	11.3 ± 7.9	11.7 ± 7.9	0.220
AMH (ng/mL)	2.3 ± 2.3	2.4 ± 2.0	0.473
Cause of infertility (%)			0.082
Uterine factor	10.6 (43/408)	12.8 (55/429)	
PCOS	7.6 (31/408)	4.9 (21/429)	
DOR	16.4 (67/408)	13.0 (56/429)	
Endometriosis	4.4 (18/408)	5.4 (23/429)	
Male factor	51.7 (211/408)	51.7 (222/429)	
RIF	5.4 (22/408)	8.4 (36/429)	
RSA	1.2 (5/408)	2.6 (11/429)	
Unexplained	2.7 (11/408)	1.2 (5/429)	

BMI, body mass index; AFC, antral follicle count; AMH, anti-Müllerian hormone; PCOS, polycystic ovary syndrome; DOR, diminished ovarian reserve; RIF, repeated implantation failure; RSA, repeated spontaneous abortion. *p*-values were calculated using Student’s *t*-test, especially Chi-square tests in infertility etiology. No statistically significant differences were observed between groups (*p* > 0.05).

**Table 2:** Effect of caffeine treatment on embryo developmental progression per patient. Data are presented as median (interquartile range) values. *p* values were calculated using the Mann–Whitney U test (two-tailed). Statistical significance is indicated as * *p* < 0.05 and ** *p* < 0.01 compared with the control group.

Outcome	Control (n = 429)	Caffeine-Treated (n = 408)	*p*-Value
2PN (%)	100.0 (41.5–100.0)	100.0 (50.0–100.0)	0.345
GQE (%)	100.0 (0.0–100.0)	100.0 (47.5–100.0)	0.011 *
Blastocyst (%)	0.0 (0.0–0.0)	0.0 (0.0–100.0)	0.006 **

**Table 3:** Mean of time points by caffeine treatment.

	Control				Caffeine-Treated			
Variables	MII from GV	MII from MI	In Vivo MII	*p*-Value	MII from GV	MII from MI	In Vivo MII	*p*-Value
**tPNf**	28.7 ± 16.7	28.3 ± 13.6	28.9 ± 10.6	0.655	30.2 ± 12.3	26.2 ± 4.0	26.0 ± 4.7	0.024 *
**t2**	32.7 ± 16.9	30.7 ± 10.4	32.0 ± 11.7	0.947	33.0 ± 7.2	32.2 ± 6.2	30.4 ± 6.7	0.133
**t3**	34.6 ± 7.4	41.2 ± 8.2	40.3 ± 9.4	0.114	39.7 ± 8.7	42.9 ± 14.2	39.5 ± 8.0	0.465
**t4**	36.7 ± 7.5	42.1 ± 8.2	43.1 ± 8.8	0.110	42.8 ± 9.7	46.7 ± 17.8	41.3 ± 7.7	0.201
**t5**	41.9 ± 8.6	47.6 ± 9.3	54.2 ± 12.1	0.011 *	50.4 ± 11.6	55.8 ± 21.6	52.3 ± 10.0	0.435
**t6**	46.8 ± 8.7	50.1 ± 6.6	57.4 ± 11.9	0.023 *	54.2 ± 12.3	59.9 ± 21.9	55.8 ± 10.2	0.431
**t7**	52.0 ± 12.5	56.8 ± 12.8	61.5 ± 11.0	0.094	59.8 ± 10.8	68.3 ± 25.6	59.3 ± 11.3	0.132
**t8**	57.3 ± 13.9	61.6 ± 10.5	64.6 ± 11.2	0.267	64.5 ± 12.7	72.0 ± 27.9	63.6 ± 12.5	0.273
**t9**	65.9 ± 8.2	76.1 ± 4.3	80.4 ± 11.4	0.006 *	75.7 ± 15.3	83.3 ± 30.7	79.1 ± 10.4	0.427
**tSC**	81.5 ± 11.0	85.1 ± 3.6	91.0 ± 10.4	0.090	83.5 ± 10.5	95.7 ± 22.6	91.5 ± 9.9	0.023 *
**tM**	88.4 ± 9.1	93.1 ± 3.1	98.1 ± 11.3	0.115	94.4 ± 10.0	109.9 ± 14.6	98.6 ± 10.8	0.046 *
**tSB**	99.3 ± 9.3	101.5 ± 2.9	105.5 ± 11.6	0.430	106.5 ± 8.3	116.8 ± 12.4	108.9 ± 11.5	0.252
**tB**	109.0 ± 12.5	107.5 ± 6.2	115.5 ± 13.7	0.482	118.8 ± 14.5	118.2 ± 1.2	116.5 ± 13.1	0.854
**tEB**	118.0 ± 0.7	124.6 ± 8.6	131.6 ± 12.9	0.093	124.9 ± 12.6	127.4 ± 7.8	128.7 ± 14.5	0.850

Data are presented as mean ± SD and analyzed by one-way ANOVA including *p*-values. * p-values < 0.05.

## Data Availability

The original contributions presented in this study are included in the article. Further inquiries can be directed to the corresponding author.

## Abbreviations

The following abbreviations are used in this manuscript:

r-IVM	Rescue in vitro maturation
IVF	In vitro fertilization
AMA	Advanced maternal age
DOR	Diminished ovarian reserve
ART	Assisted reproductive technology
MPF	Maturation-promoting factor
MPAK	Mitogen-activated protein kinase
ICSI	Intracytoplasmic sperm injection
COCs	Cumulus-oocyte complexes
GQE	Good-quality embryo
ICM	Inner cell mass
TE	Trophectoderm
AMH	Anti-Mullerian hormone
AFC	Antral follicle count

## References

[1] Fauser B., Adamson G.D., Boivin J., Chambers G.M., de Geyter C., Dyer S., Inhorn M.C., Schmidt L., Serour G.I., Tarlatzis B. (2024). Declining global fertility rates and the implications for family planning and family building: An IFFS consensus document based on a narrative review of the literature. Hum. Reprod. Update.

[2] Liu X., Zhao Y., Feng Y., Wang S., Zhang J. (2025). Ovarian Aging: Mechanisms, Age-Related Disorders, and Therapeutic Interventions. MedComm.

[3] Wang T., Xu P., Yuan J., Chen H., Guo X., Gao J., Wang Y., Yao D., Li X., Liu B. (2025). Mitochondrial dysfunction in oocytes: Implications for fertility and ageing. J. Ovarian Res..

[4] Hardy M.L.M., Day M.L., Morris M.B. (2021). Redox Regulation and Oxidative Stress in Mammalian Oocytes and Embryos Developed In Vivo and In Vitro. Int. J. Environ. Res. Public Health.

[5] Mandelbaum R.S., Awadalla M.S., Smith M.B., Violette C.J., Klooster B.L., Danis R.B., McGinnis L.K., Ho J.R., Bendikson K.A., Paulson R.J. (2021). Developmental potential of immature human oocytes aspirated after controlled ovarian stimulation. J. Assist. Reprod. Genet..

[6] Lu Y., Ferrer-Buitrago M., Popovic M., Neupane J., De Vos W.H., Lierman S., Van den Abbeel E., Van der Jeught M., Nikiforaki D., De Sutter P. (2018). Patients with a high proportion of immature and meiotically resistant oocytes experience defective nuclear oocyte maturation patterns and impaired pregnancy outcomes. Reprod. Biomed. Online.

[7] Soler N., Cimadomo D., Escrich L., Grau N., Galan A., Alama P., de Los Santos M.J., Rienzi L., Escriba M.J. (2025). Rescue in vitro maturation of germinal vesicle oocytes after ovarian stimulation: The importance of the culture media. Hum. Reprod..

[8] Wang Y., Zhang Y., Li T., Ren Y., Zhou P., Fu L., Xiao C., Huang Z., Huang H., Xie W. (2025). Transcriptional insights on the incomplete cytoplasmic maturation and developmental potential of oocytes cultured without granulosa cells in mice. BMC Genom..

[9] Bogliolo L., Ledda S., Leoni G., Naitana S., Moor R.M. (2000). Activity of maturation promoting factor (MPF) and mitogen-activated protein kinase (MAPK) after parthenogenetic activation of ovine oocytes. Cloning.

[10] Galeska E., Kowalczyk A., Wrzecinska M., Garcia M.C., Czerniawska-Piatkowska E., Gwozdziewicz S., Witkiewicz W., Dobrzanski Z. (2025). The Importance of Mitochondrial Processes in the Maturation and Acquisition of Competences of Oocytes and Embryo Culture. Int. J. Mol. Sci..

[11] Xu X., Pang Y., Fan X. (2025). Mitochondria in oxidative stress, inflammation and aging: From mechanisms to therapeutic advances. Signal Transduct. Target. Ther..

[12] Marcinek K., Luzak B., Rozalski M. (2024). The Effects of Caffeine on Blood Platelets and the Cardiovascular System through Adenosine Receptors. Int. J. Mol. Sci..

[13] Dorvigny B.M., Tavares L.S., de Almeida I.A., Santana L.N., de Souza Silva E., de Souza J.K.U., Soares A.F., da Silva Junior V.A., Lima-Filho J.V. (2022). Antiinflammatory and antiinfective effect of caffeine in a mouse model of disseminated salmonellosis. Phytother. Res..

[14] Epplen A.S.C., Rothoft M., Stahlke S., Theiss C., Matschke V. (2025). Caffeine mitigates ROS accumulation and attenuates motor neuron degeneration in the wobbler mouse model of amyotrophic lateral sclerosis. Cell Commun. Signal.

[15] Osz B.E., Jitca G., Stefanescu R.E., Puscas A., Tero-Vescan A., Vari C.E. (2022). Caffeine and Its Antioxidant Properties-It Is All about Dose and Source. Int. J. Mol. Sci..

[16] Lee J.H., Campbell K.H. (2008). Caffeine treatment prevents age-related changes in ovine oocytes and increases cell numbers in blastocysts produced by somatic cell nuclear transfer. Cloning Stem Cells.

[17] Ha J., Kim S., Jang H., Ko Y.R., Han S., Lee W.S., Eum J.H. (2026). Caffeine Supplementation Enhances Aged Human Oocyte Quality and Embryo Development in In Vitro Fertilization: A Retrospective Paired Study. Reprod. Med. Biol..

[18] Bernal-Ulloa S.M., Lucas-Hahn A., Herrmann D., Hadeler K.G., Aldag P., Baulain U., Niemann H. (2016). Oocyte pre-IVM with caffeine improves bovine embryo survival after vitrification. Theriogenology.

[19] Kim G., Roy P.K., Fang X., Hassan B.M., Cho J. (2019). Improved preimplantation development of porcine somatic cell nuclear transfer embryos by caffeine treatment. J. Vet. Sci..

[20] Zhang N., Wakai T., Fissore R.A. (2011). Caffeine alleviates the deterioration of Ca^2+^ release mechanisms and fragmentation of in vitro-aged mouse eggs. Mol. Reprod. Dev..

[21] Tachibana M., Amato P., Sparman M., Gutierrez N.M., Tippner-Hedges R., Ma H., Kang E., Fulati A., Lee H.S., Sritanaudomchai H. (2013). Human embryonic stem cells derived by somatic cell nuclear transfer. Cell.

[22] Yang Q., Zhu L., Wang M., Huang B., Li Z., Hu J., Xi Q., Liu J., Jin L. (2021). Analysis of maturation dynamics and developmental competence of in vitro matured oocytes under time-lapse monitoring. Reprod. Biol. Endocrinol..

[23] Ciray H.N., Campbell A., Agerholm I.E., Aguilar J., Chamayou S., Esbert M., Sayed S., Time-Lapse User G. (2014). Proposed guidelines on the nomenclature and annotation of dynamic human embryo monitoring by a time-lapse user group. Hum. Reprod..

[24] Conti M., Franciosi F. (2018). Acquisition of oocyte competence to develop as an embryo: Integrated nuclear and cytoplasmic events. Hum. Reprod. Update.

[25] Mao L., Lou H., Lou Y., Wang N., Jin F. (2014). Behaviour of cytoplasmic organelles and cytoskeleton during oocyte maturation. Reprod. Biomed. Online.

[26] Baldini G.M., Ferri D., Malvasi A., Lagana A.S., Vimercati A., Dellino M., Baldini D., Trojano G. (2024). Genetic Abnormalities of Oocyte Maturation: Mechanisms and Clinical Implications. Int. J. Mol. Sci..

